# Prioritarian principles for digital health in low resource settings

**DOI:** 10.1136/medethics-2019-105468

**Published:** 2020-01-16

**Authors:** Niall Winters, Sridhar Venkatapuram, Anne Geniets, Emma Wynne-Bannister

**Affiliations:** 1 Department of Education, University of Oxford, Oxford, UK; 2 Department of Global Health and Social Medicine, King’s College London, London, UK

**Keywords:** information technology, public health ethics, health workforce, distributive justice, philosophical ethics

## Abstract

This theoretical paper argues for prioritarianism as an ethical underpinning for digital health in contexts of extreme disadvantage. In support of this claim, the paper develops three prioritarian principles for making ethical decisions for digital health programme design, grounded in the normative position that the greater the need (of the marginalised), the stronger the moral claim. The principles are positioned as an alternative view to the prevailing utilitarian approach to digital health, which the paper argues is not sufficient to address the needs of the worst off. As researchers of digital health, we must ensure that the most globally marginalised are not overlooked by overtly technocentric implementation practices. Consequently, the paper concludes by advocating for use of the three principles to support stronger critical reflection on the ethics involved in the design and implementation of digital health programmes.

## Introduction

Digital health technologies are increasingly visible in the healthcare systems of low-income and middle-income countries (LMICs). There is no consensus yet among experts regarding the precise definition of digital health. The WHO’s first guidelines on digital health states the term was introduced as ‘a broad umbrella term encompassing eHealth (which includes mHealth), as well as emerging areas, such as the use of advanced computing sciences in “big data,” genomics and artificial intelligence’.^1^ Digital health examples include using mobile phones to help train community healthworkers,^2^ digital referrals^3^ and mobile clinical decision support systems.^4^ While digital health has seen a large and growing body of implementation research in LMICs, the ethical issues it raises have also motivated research interest, although to a lesser extent.^5^ However, this research has largely focused on describing the ethics implicated in the practical deployment of digital health technologies. Issues include the ethics of anonymity, data collection methods, data retention and use, and informed consent.^6 7^ Some authors have also developed normative ethical frameworks and guidelines for digital health implementation.^8 9^Although this growing area of research is valuable, it does not cover the breadth of ethical issues related to digital health.

We contend that ethics of digital health is urgently required to study, manage or mitigate the structural neglect and digital inequalities that could result from the use of digital health in low resource settings. Compelling evidence from other fields including sociology, anthropology and peace studies is clear about the impact of structural neglect on the socially worst off in society. This phenomenon has been termed ‘structural violence’^10 11^ and refers to the structural and institutional constraints placed on (marginalised) people, which prevent them from living the life they value. Any social structure, be it economic, political, educational or cultural, which prevents individuals from expressing themselves and developing their full potential, can accordingly be conceptualised as structural violence. The field of social medicine has examined how this applies to healthcare, including the impact of inequality on longevity.^12^ Studies that have examined digital inequalities have also clearly evidenced the negative impact of technological innovation and implementation on the worst off.^13^

Our aim in this theoretical paper is twofold. First, we argue for particular ethics of digital health for low resource settings, namely prioritarianism, that is, giving priority to the needs of the socially worst off. We argue that it offers a feasible and better alternative to utilitarianism, which we contend is pervasive in global health and in programmes using digital health in LMICs.^14^ Second, in light of the shortcomings of approaches that seek to achieve Millennium Development Goals (MDGs) targets without concern for equity,^15–17^ the current Sustainable Development Goals (SDGs) agenda is frequently linked with the commitment of ‘leaving no one behind’. Critically, prioritarianism provides the ethical framework for such rhetorical commitment; it motivates addressing the needs of the worst off and grounds it in fundamental and globally shared values, such as equality. Fundamental and globally shared values are important in moral philosophy. Scanlon argues for the plausibility of such shared moral principles as those that could not be reasonably rejected.^18^ Such a position has found empirical validation in a recent global survey that quantified ‘societal expectations about the ethical principles that should guide machine behaviour’. The first of its kind large-scale documentation of moral decisions has improved our understanding of the shared and divergent ethical values through simulation of driverless car accidents.^18 19^ In reflecting the global shared value of equality and, plausibly, helping the worst off, we propose three principles, derived from prioritarianism, as a practical tool that can be applied to digital health programme design, development and implementation.

### Prioritarianism: in brief

Prioritarianism is one approach to distributive justice—the ethical determination of important moral goods for human beings and their well-being, as well as the distribution rules for these moral goods. Different theories of distributive justice differ regarding what they identify as a moral good, who is included and excluded in the distribution, and the distribution rules. The ‘equality of what’ debates have been flourishing for a few decades spurred on by Rawls’ Theory of Justice^20 21^ and are now well-known and widely debated.^22^ In the first instance, prioritarianism is about the distribution rules, which could be potentially shared across diverse approaches. According to Holtug, prioritarianism expresses giving ‘priority to the worse off in the distribution of advantages’.^23^ (This is distinct from utilitarianism, which asserts that any act or intervention should achieve ‘the greatest good for the greatest number of people’.^24^ Distribution of a valuable good or service should happen in such a way as to maximise well-being within a society overall.)

The development of prioritarian reasoning has been profoundly influenced by the arguments of Parfit.^25^ He provided an example (taken from Nagel)^26^ to illustrate how it works and why it is more coherent than alternatives: a father has two children, one of whom has a disability. If the father moves to the city, the child with the disability will benefit from specialist care. However, if he moves to a suburb instead, the child who is not disabled will flourish, and benefit more than the child with a disability. Nagel provides certain reasons for why the father should decide to move to the city to avail the specialist care for his child with disabilities. Parfit points out that ‘Nagel’s decision turns on the relative importance of two facts: he could give one child a greater benefit (ie, maximise goods), but the other child is worse off (absolutely and relative to what he could have been).^25^ In deciding to move to the city, the father is *prioritising* the needs of the child with a disability over the other child’s benefits. Furthermore, and importantly, the choice results in a ‘smaller sum of benefits (overall), for the sake of a better distribution’.^25^ Better distribution, or more equity, stands in contrast with the utilitarian aim of maximising total benefits. Temkin presents an insightful discussion on distribution rules and patterns.^27^ However, the moral justification of tolerating lower overall benefits (eg, numbers) for the sake of better distribution of benefits to the worst off is the core of prioritarianism.

Stated more formally, *axiological prioritarianism*, is defined by Holtug as follows:

an outcome is noninstrumentally better, the larger the sum of weighted individual advantages it contains, where advantages are weighted such that they gain a greater value, the worse off the individual is to whom they accrue.^23^

That is, equity weights added to the benefits of the worse off produce a greater sum overall than the sum of just total benefits. Those who do not recognise equity weights would just see a greater sum of benefits foregone for the sake of fewer benefits. It may help to illustrate with a different example: the first dollar is more valuable for a person who has no money than the 99th dollar is to a person who already has 98 dollars. Each may be holding one equal dollar, but the additional value (‘advantage’) the first one dollar has to the person with no money is of moral importance and given weight. Consequently, the distribution of 98:1 is better than 99:0 because the advantage gained/disadvantage alleviated by the first 1 unit is greater than the advantage gained/disadvantage removed by the 99th unit. This is not an empirical assertion—such as the case of the first unit of food being more impactful than the 99th unit for a starving person. It is a moral assertion about advantages and disadvantages. In an acute situation where two are disadvantaged, the situation would be better where the worst off becomes better off. The principles outlined later in this paper are a practical means of doing this within the context of digital health.

Barsdorf and Millum note that the ‘idea that the worst off deserve greater priority is widely endorsed in medical ethics and philosophy’.^28^ Although that may be understandable in emergency clinical care and academic philosophy, in public health and global health, the prevailing view is not prioritarian. Furthermore, prioritarianism in medical ethics and care, or helping the worst off in a healthcare setting, is fundamentally motivated by instrumental reasons—the prevention of death or serious harm. In non-instrumental prioritarianism such as the above-mentioned money example, 98:55 would be a better distribution than 99:54, irrespective of the consequences of disadvantage. That is, disadvantage, such as constrained well-being, is intrinsically a morally bad thing.

We assert that a utilitarian or maximising ethos is dominant in global health. In support of this claim, we highlight two key long-standing trends:

1. In global health, the rational and right action is that which produces the greatest good (ie, numbers vaccinated, cases averted, DALYs reduced and so on), where national and global health targets are pervasive, underlining a maximising ethos.
2. Scaling-up technologies are often prioritised over-reaching the worst off and the distribution of health innovations is not done in a just way.^29^ In mHealth in particular, the focus on the scale is well recognised, and the development of a ‘robust evidence base for the scale-up of mHealth interventions’ is a vibrant area of research.^30^ The importance of scale is also evidenced in the ‘Principles for Digital Development’.^31^ These principles are widely considered the template by which major donors in the international development frame and understand the design and development of technology in LMICs. Within the principles, a focus on the scale is clearly evident, for example, ‘Designing for scale means thinking beyond the pilot and making choices that will enable widespread adoption later, as well as determining what will be affordable and usable by a whole country or region, rather than by a few pilot communities’. Here, we see that the utilitarian maxim of doing the greatest good for the greatest number of people is achieved by digital health interventions that will enable the ‘widespread adoption’ of ‘affordable and usable’ technologies across ‘a whole country or region’.

However, against the prevailing utilitarian and maximising ethos of global health, there has been a prominent and long-standing prioritarian approach of addressing the disadvantages faced by the worse off. Farmer, one of its most vocal advocates and practitioners, expresses it in the principle of providing a ‘preferential option for the poor’.^32^ Farmer states that this means ‘medicine has a clear—if not always observed—mandate to devote itself to populations struggling against poverty’.^32^ This approach to global health medicine, in turn, owes its ethical stance in large parts to the radical pedagogical and theological liberation movements originating in Latin America in the late 1950s and 1960s. These movements focused on the poor (most socially disadvantaged) and their liberation (of the oppressed).^33–36^ Prioritarianism, with various kinds of justifications, has had a long history and is visible in societies across the world. It has also been influential in contemporary development economics, and development studies and programmes. But it has not yet made significant inroads to ‘health development’ or global health, or, indeed, in digital health programmes in LMICs. This should be surprising given the rhetoric of digital health being the panacea for the poor health of the worst off in the world. Prioritarian principles would be more in line with the rhetoric.

## Digital health and the worst off

The disconnect between global and local agendas is an active area of research.^37^ Given the disconnect between the SDGs and Universal Health Coverage (UHC) vs the local realities facing the worst off people on a day-to-day basis, more research is required to understand how and why technologies and innovations are not reaching those who need them the most. Undoubtedly, the complexities of implementation and delivery make providing access to digital health to the worst off challenging. However, it may also be that the underlying ethical positions of digital health programmes are perpetuating or worsening digital inequities. For instance, as noted above, scaling-up, and fast, is often prioritised over-reaching the worst off. That is, getting more users is valued more than thoughtfully considering different distribution patterns of technology that could increase the positive impact of digital health on the lives of the worst off.

Despite the pervasive rhetoric of helping the worst off through using digital health in low resource settings, the utilitarian reasoning of maximising impact or uptake does not necessarily or often include the worst off. This was clearly shown to be the case and problem with the singular focus on achieving targets of the MDG agenda^38^ The goals of scaling up versus helping the worst off often pull in different directions. To take a simple example, reaching poor girls and women confined to their homes requires more financial and human resources than reaching men in public spaces or workplaces. For the same amount of resources, more male cases could be averted than female cases. The same could be said for two different socioeconomic groups, or other social groups stratified by disadvantage. Prioritarianism offers a viable alternative to utilitarian reasoning in digital health that works in the interests of the worst off because it is distribution sensitive, that is, it is ‘sensitive to the way in which advantages are distributed among individuals rather than just concerned with the overall level or sum of them’.^23^

Prioritarianism also provides an alternative view of technology, one that sees it as socially embedded in its context of use. We argue that prioritarianism could avoid or overcome some of the theoretical, methodological and empirical weaknesses inherent in utilitarianism as an ethical foundation for digital health. The need for this alternative prioritarian view is clear given the persistent lack of access to health technologies for those who require them most. Sinha and Barry have argued that this lack is most clearly apparent in LMICs.^39^ And Farmer claims more broadly that there is a continuous failure to distribute health innovations in a just way, while increasing disparities in health outcomes.^29^ Repeatedly over 4 years, the WHO Global Forum on Medical Devices emphasised the importance of accessibility and the role of technology in realising UHC, in line with their Health for All priority.^40^

### How can digital health support the needs of the worst off?

In order to support these prioritarian intuitions and facilitate practical reasoning in programme design, development and implementation of digital health, we offer a set of three principles. The principles were developed for two main reasons. First, there is a need to offer an alternative to the dominant approach to digital health in LMICs of first focusing on those who are ‘easiest to reach’, as distinct from the ‘seldom-heard’. The needs of the most marginalised are often the last to be addressed; their voice is lost to those in a more privileged positions and those who are valued as early adopters. Second, one of the key failings of a utilitarian positioning to digital health (or any innovation) is that it does not sufficiently recognise ‘the complex, networked and learning-intensive features of technology’,^41^ or the social and contextual meaning of technology^42^ and is overly concerned with scale.^30^

If such an understanding is not urgently integrated into programmes, structural neglect and digital inequalities could result from the use of digital health in low resource settings. Our response has been to develop the principles as a useful reasoning tool by which necessary consideration is given to how digital health programmes can be weighted in favour of the worst off, especially during the early stages of their design and implementation. These principles should be viewed as evolving and are intended as a baseline for discussion. The primary aim is to support the processes of working through how to embed a prioritarian ethical framing when using digital health and technology in development more broadly. However, while we focus on digital health, we believe the prioritarian principles could have wider applicability to other domains of global health and development.

## The principles

The three principles were developed at two Wellcome Trust funded workshops in Oxford and London. Seventeen participants from academia (health, education, development, philosophy and technology) and international non-governmental organisations (INGOs) discussed digital health case studies from India, East and West Africa, appropriate theoretical conceptualisations and philosophical positions, and suitable methodological and empirical approaches. Presenters and discussants offered reflections on what worked (and what did not) and why, drawing on experience from their own empirical contexts. Discussions always included ways in which ethics is (or is not) embedded within discourses in technology-related discussions within each sector. The importance of focusing on structural inequalities was emphasised, particularly in contexts where the latest technologies were sometimes seen by funders as ‘leap-frog’ solutions to long-standing and complex structural problems. Aspects of scale and scaling-up were also problematised: the assumption should not be that once you have designed for the worst off in one context, these technologies can be scaled for different contexts, as two groups of the worst off can have substantial differences between them. The analysis and principles were presented and further developed from feedback received from a presentation at the 2017 Oxford Global Health and Bioethics International Conference.

### Principle 1: identify what constitutes disadvantage so the social value of digital health can be maximised for the worst off

Barsdorf and Millum discuss how the social value of a project can be determined by its benefit to marginalised populations: ‘the more disadvantaged the beneficiaries of a research project, the higher its social value’.^43^ However, in order to determine social value, we need to first identify what constitutes disadvantage. Prioritarianism relies on the ability to determine who is the worst off so that differences in distribution can be accounted for in the allocation of resources (money, time, technology and so on). Sharp and Millum offer one way to determine who are the worst off.^44^ One core component of their argument, which is relevant to our discussion here, is their review of the different approaches to conceptualising disadvantage and their objects of value (moral goods, advantages or ‘currencies’ in their terminology). They acknowledge that developing ‘an operational measure’ of disadvantage is a significant challenge because of the ‘the long-running philosophical disagreement about the currency of advantage’.^44^ That is, in addition to what the distribution rules should be, there is also profound disagreement about the valuable good to be distributed: ‘[r]esourcists, welfarists and proponents of the capabilities approach all characterise advantage in different ways’.^44^ Sharpe and Millum reach the interesting conclusion that there are identifiable common constituents of disadvantage across the different moral goods:

…there is considerable consensus about a core set of indicators that track advantage, however defined. For instance, it is widely agreed that education, minimal levels of material wellbeing, mental and physical health, and the protection of certain civil and political rights are all very important to ensuring the opportunity for a flourishing life. Thus, for practical purposes, those who lack access to education, have a low material standard of living, face severe health problems, and whose rights are not protected, are very likely to belong among the worst off.^44^

Their discussion is helpful in so far as it at least begins to identify how to distinguish who is worst off. Moreover, the review of various approaches to distributive justice shows that there are substantial and active bodies of research helping to identify the worst off on which prioritarianism in digital health can build. The principle itself is agnostic about *the form* of disadvantage. That is, the worst off do not have to be worst off in terms of the holdings of the good to be distributed. They can be worst off in other forms of disadvantage.

### Principle 2: the design and implementation of digital health should address the needs of the worst off through their participation

Once the worst off have been identified, and/or social gradient in disadvantage determined, the second principle focuses on determining how best digital health can address their needs. This requires developing a deep understanding of the relationship between technology and the intersectional disadvantage the worst off face on an everyday basis. Technologists have not done a great job alleviating complex disadvantages that have produced healthy scepticism regarding the potential impact of technology on the lives of the worst off.^45^ Principle 2 asserts that digital health *can and should* address the needs of the worst off, but only when technology design and development involves the worst off in the design process in a meaningful way.^46^ The principle makes sure that technological developments addressing their needs are identified, addressed and integrated from the start. This principle draws on substantive research in social justice (eg, Farmer’s ‘preferential option for the poor’,^32^ Fraser’s conceptualisation of ‘parity of participation’,^47^ with the relatively small but growing community of Human Computer Interaction for Development (HCI4D) researchers and practitioners^48^ and on research in various forms of Participatory Action Research (PAR).^46^ Through a process that reflects the principle, the form of digital health intervention can be specified iteratively in increasingly higher fidelity and directly related to its function of benefiting the worst off.

A helpful illustrative example comes from our research on the co-development of a mobile-based training programme for community health workers (CHWs) in Kenya starting in 2013. To address the needs of the worst off in their community—in this case, children with disabilities and their mothers—smartphones were chosen as the technology, rather than the far more available low-end phones. The participation of CHWs in designing the intervention was critical to this technology decision. Their stated need to be able to assess the health and social care needs of children in their community required an app with smartphone-enabled functionalities (rather than a low-end phone). Thus, by taking a priority view, we rejected a utilitarian, ‘top-down’ approach to technology design and implementation that would have meant *not* working to overcome the intersectional disadvantage faced by the children with disabilities and their mothers and instead would have focused on an existing priority area.^49^ We emphasise that the rationale behind using smartphones as part of the programme intervention was not utilitarian or maximising reach for participants. Instead, the focus was on a training and decision-making app that was co-designed to directly meet the needs of the CHWs and the mothers of children with disabilities. Simply using the most available option of low-end phones would not have offered the best digital health intervention possible, given their limited functionality.

This reasoning has parallels with Farmer’s and Gutierrez’s concept of accompaniment. Accompaniment for them is listening to the poor and the oppressed and walking with them, ‘thinking, living, working and reflecting with the people who know most about poverty’.^50^ In terms of a programme design in digital health, the ways in which technology can be embedded to support outreach (through improved communication and peer decision-making, eg) is critical and remains under-researched. This principle supports this process through the exploration of the following questions as part of any design process:

- How can marginalised groups have a role in the design and roll-out of new technologies?
- How can an equitable environment for these developments be intentionally promoted?
- How can marginalised groups be supported to identify culturally relevant and contextually appropriate interventions?
- Are the most disadvantaged within the disadvantaged participating and voicing their needs?

### Principle 3: be ethically reflective about our (global) ethical obligations as (global) digital health researchers and practitioners

Identifying the most disadvantaged (Principle 1) and prioritising the voices and needs of the marginalised in the design, development implementation and evaluation of digital health technologies (Principle 2) leads to the third ethical principle of assessing our obligations as researchers and practitioners. The hard truth of global and digital health is that those with power (eg, funders, practitioners and researchers) must provide adequate resources if they are serious about aligning themselves with prioritarianism when addressing the needs of the worst off. This implies that those with power must go beyond their usual day-to-day interactions with people who are marginalised to become *advocates* for them.^32^ Instead of relying on an ethical stance that only describes and manages social inequality (which has merits in itself), the priority view transforms those with power into advocates for social change by effectively tackling the social and political structures that produce and perpetuate inequality. That is, a multitude of intersecting political, social, cultural and economic processes and forces often conspire to constrain the agency of those people who are most disadvantaged.^32^ Raising their well-being is not a passive or minor effort. It is an effort that militates against the injustice of status-quo prevailing inequalities of advantage or well-being.

Let us take an example from our previous work investigating existing research on the supports available for abused children with disabilities in East Africa^49^. In order to motivate the need to help those at the bottom first, from a research perspective, it was necessary to map the types of evidence that exist on this topic. Our scoping review and evidence map documented the coverage, patterns and gaps in existing research on the abuse of children with disabilities in East Africa. In doing this, we showed that the abuse of children with disabilities—a highly marginalised group—was a topic grossly neglected that must be prioritised. This research provided the necessary evidence to underpin and develop a strategic agenda in this area to better determine *what* future research in order to undertake to address this pressing lacuna. In some ways, this involved us assessing our obligations as researchers. One key question we faced was how can this be achieved in a pragmatic way that allows us to promote the voices of the worst off? To address this question, we drew on the research of Sarradon-Eck *et al* on caring on the margins of the healthcare system.^51^ Through their work, they emphasise the necessity to go beyond immediate improvements to healthcare for the worst off and change the environment to support them to regain their human rights, their access to social protection, and ultimately, their autonomy and social identity as participating members of society. They point out the onus is on projects to reach out to the worst off, *not* on those in need of care to seek it.

Principle 3 can be applied at the systems level, where we are often dealing with people who are not only marginalised but also highly stigmatised (eg, mothers of children with disabilities). Sarradon-Eck *et al* point out how this may lead some doctors and nurses in the formal health system to view the marginalised with ‘a form of symbolic contagion from the stigma’ arising from their situation.^29^ This is important to take into consideration when addressing the social factors that impact on the integration of digital health programmes aimed at the marginalised into the wider health system. Reflecting on our role as researchers and practitioners, this brings up the absolute need to attend to issues of power, representation and culture.^30^ Asking ethically reflective questions of how project interactions may have created misunderstandings, how to ensure all participants have the opportunity to take part and recognising the ways in which interactions can be ‘subtly oppressive’ and potentially reinforcing power, particularly in cases where technology is new to participants. Providing opportunities to elicit feedback to make adjustments to any instructional design, including knowledge co-construction can be a critically important means of resisting this.

## Conclusions

The three principles presented in this paper offer a tool and a starting point for discussion of how to take a priority view on the design, development and implementation and evaluation of digital health in LMICs. They are both conceptual and pragmatic, offering a means by which the needs of the worst off can have a central role in programme development. The principles are a way to move away from the current utilitarian position in digital health. As we have made clear, the currently prevailing approach is problematic for the worst off but it can be addressed by an ethic of prioritarianism that also deeply embeds participation of the marginalised in programmes that are designed from an understanding of the sociotechnical complexities of implementation. These principles are meant to be continually evolving alongside technology, developed from experiences of previous digital health projects in LMICs and focused discussions among relevant experts. We welcome efforts to apply them systematically and to further discussions to help improve and add to them.
